# Facile Synthesis and Fabrication of *NIPAM*-Based Cryogels for Environmental Remediation

**DOI:** 10.3390/gels9010064

**Published:** 2023-01-12

**Authors:** Jaweria Ambreen, Abdul Haleem, Aqeel Ahmed Shah, Fozia Mushtaq, Muhammad Siddiq, Muhammad Ali Bhatti, Syed Nizam Uddin Shah Bukhari, Ali Dad Chandio, Wael A. Mahdi, Sultan Alshehri

**Affiliations:** 1 Department of Chemistry, COMSATS University Islamabad, Park Road, Islamabad 45550, Pakistan; 2 Department of Chemistry, Quaid-i-Azam University, Islamabad 45550, Pakistan; 3 School of Chemistry and Chemical Engineering, Jiangsu University, Zhenjiang 212013, China; 4 Wet Chemistry Laboratory, Department of Metallurgical Engineering, NED University of Engineering and Technology, University Road, Karachi 75720, Pakistan; 5 Institute of Environmental Sciences, University of Sindh, Jamshoro 76080, Pakistan; 6 Department of Basic Science and Humanities, Dawood University of Engineering and Technology, Karachi 74800, Pakistan; 7 Department of Pharmaceutics, College of Pharmacy, King Saud University, Riyadh 11451, Saudi Arabia

**Keywords:** cryogel, catalysis, reduction, dye, degradation, antibacterial activities

## Abstract

Herein, polymeric cryogels containing poly(N-isopropylacrylamide) were synthesized by cryo-polymerization at subzero temperature. The synthesized cryogels were loaded with silver and palladium nanoparticles by the chemical reduction method at room temperature using the reducing agent NaBH_4_. Moreover, for comparison with cryogels, pure poly(N-isopropylacrylamide) hydrogel and its silver hybrid were also prepared by the conventional method at room temperature. The chemical structure and functional group analysis of the pure cryogels was confirmed by Fourier transform infrared spectroscopy. The synthesis of hybrid cryogels was confirmed by the X-ray diffraction technique and energy dispersive X-ray. The pore size and surface morphology of the pure cryogels, their respective hybrid cryogels and of conventional hydrogels were studied by using the scanning electron microscopy technique. The hybrid cryogels were successfully used as a catalyst for the degradation of methyl orange dye. The degradation performance of the hybrid cryogels was much better than its counterpart hybrid hydrogel for methyl orange dye. The effect of temperature and amount of catalyst on catalytic performance was studied by UV-visible spectroscopy. The reduction follows pseudo-first-order reaction kinetics. In addition, the antibacterial activities of these cryogels were evaluated against Gram-positive bacteria (*Staphylococcus aureus*, ATCC: 2593) and Gram-negative bacteria (*Escherichia coli*, ATCC: 25922). Both hybrid cryogels have shown much better antibacterial activity for these two strains of bacteria compared to pure cryogels. The results indicate that these cryogels are potential candidates for water purification systems as well as biomedical applications.

## 1. Introduction

Industrial effluents are one of the major causes of water pollution, triggering serious environmental issues across the globe [1]. The adverse effect of contaminated water is reducing the average life expectancy on earth [2]. Therefore, it is highly desirable to adopt new strategies to efficiently remove the toxins from water, ameliorate its quality and make it available for use. There have been tremendous attempts to design new materials with improved performance for removal of toxic substances from water bodies; but there is still a demand for some more efficient materials [3,4]. Among various approaches, adsorption is one of the most efficient ways to remove these harmful pollutants from water [5,6]. Diverse types of catalytic adsorbents have been fabricated for the degradation of several water-soluble organic dyes and nitro compounds; frequently used in textile industries [7,8]. For instance, multiple studies show that metal nanoparticles are presumed to exhibit good catalytic potential owing to their high surface-to-volume ratio [9,10,11]. However, this greater surface area and weaker intermolecular forces cause the clustering of metal nanoparticles in an aqueous medium, hence, declining their catalytic ability.

To prevent the aggregation of metal nanoparticles, different matrices are commonly used. For example, synthetic polymeric gels have drawn significant importance due to their porous structure and facile variation of chemical structure for the removal of toxins [12,13,14]. Additionally, gels could absorb larger contents of water, thus making the aqueous catalytic reactions more feasible compared to other types of carrier systems. Gel frameworks often contain moieties such as COOH, OH, -SH, NH_2_, -SO_3_H, etc., which could serve as the metal binding sites and enable them to have a higher uptake of metal ions from aqueous solutions. An appropriate reducing agent could transform the metallic ions into nanoparticles by in situ reduction, and these nanoparticles would be embedded into the gel matrix on account of the electrostatic and ion-dipole forces of attraction [15]. Moreover, hydrogels are mostly smart materials that could respond to external chemical and physical stimuli such as temperature, pH, ionic strength, etc. Among various smart polymers, poly(N-isopropylacrylamide) has been substantially utilized for the stabilization of nanoparticles [16,17]. Yan and coworkers have incorporated a Ag-nanoparticle into the poly(N-isopropylacrylamide)-based composite microgel matrix for the evaluation of its temperature-dependent catalytic performance. It was shown that the catalytic activity of composite microgel could be tailored easily by varying the temperature [18].

Currently, cryo-polymerization has emerged as an applicable approach to synthesize inter-connected macro-porous polymeric gels to be employed as adsorbents [19]. The polymeric cryogels are a subclass of hydrogels synthesized at a sub-zero temperature. Their macro porous structure consists of two phases; a solid and an unfrozen liquid phase [20]. Explicitly, cryo-polymerization is performed at temperatures lower than the freezing point of the polymerization mixture. Here, the frozen polymerization solution initiates the formation of macro-pores in the structure; whereas the unfrozen liquid microphase acts as the polymerization site to build the framework of cryogels [21]. During the synthesis of cryogels, the cross-linking reactions take place in the non-frozen liquid phase [22]. Mostly, cryogelation is a time-consuming process; however, the cryo-concentration effect aids the polymerization process to progress at a reasonable rate even at relatively low temperatures [23,24].

Cryogels have received tremendous attention in multiple fields owing to their efficient mass transport ability, large surface area, elasticity, low density, high adsorption capacity, excellent swelling ability, quick response to external stimuli and unique morphology [25,26,27,28,29,30]. For example, super macro porous cryogels are used for drug delivery and promote the regeneration of tissues efficiently [31]. Similarly, Kudaibergenov et al. have synthesized poly (ampholyte)-based cryogels and utilized them as a matrix for the separation of bio macromolecules [32]. In addition, Kochaporn Chullasat et al. have designed a sorbent through the combination of hybrid monolith poly(pyrrole) overlayed with graphene oxide penetrated polyvinyl alcohol cryogels. This sorbent was used for detecting sulfonamides. It was reported that these sorbents exhibit a high extraction efficiency of sulphonamides and their re-useability was at least 10 times [33].

One of the main features of cryogels is their fast reaction rate compared to conventional microgels. It may be attributed to their sponge-like structure, with a greater number of pores present externally and internally furnishing more active sites to the target. Presently, the synthesis of hybrid gels under cryogenic conditions is considered a facile approach to designing efficient catalysts for contaminant removal [34,35]. In addition, an important characteristic of cryogels is their use as a matrix due to their good mechanical and chemical stability [36]. These cryogels have shown excellent catalytic activities in the incorporation of nanoparticles e.g., of cobalt, nickel, copper, silver, gold, palladium, etc., in their matrix to make their hybrid [37]. Cryogel catalysts are able to remove toxic compounds from water bodies in less time and could be employed to work in real implementations [38]. For example, Abdul Haleem et al. have fabricated poly (lauryl acrylate) containing cryogels as oil sorbents. The effect of different parameters such as temperature and the cross-linking monomer amount on the composition of cryogels was studied. The cryogels had shown high adsorption ability as oil sorbents from wastewater [39]. In another report, Abdul Haleem et al. synthesized poly(N-isopropylacrylamide-co-acrylamido-2-methylpropane sulfonic acid) hybrid cryogels and used them for the reduction of p-nitro phenol. It has been stated that the catalytic activity increases with the increasing temperature [40].

In this manuscript, we reported the synthesis of poly (N-Isopropylacryamide)-based cryogels, which were used as a matrix for the synthesis of Ag and Pd nanoparticles embedded in them to make their respective hybrids. The incorporation of Ag and Pd nanoparticles in hybrid cryogels renders them improved catalytic properties. The catalytic performance was evaluated for the degradation of methyl orange dye in an aqueous medium. The influence of different parameters such as temperature and catalyst dosage on the cryogel adsorption capacity was studied. These cryogels could potentially be used for the catalytic degradation of organic pollutants and dyes from wastewater effluents.

## 2. Experimental Procedure

### 2.1. Materials

N-isopropylacrylamide (NIPAM, 99%, J&K Chemical) (0.5 M, 0.5488 g), N,N-methylene bisacryamide (MBA, 99%, Sinopharm Chemical) (0.5 M, 0.023 g), N,N,N’,N’-tetramethylethylenediamine (TEMED, 99%, Sinopharm Chemical), ammonium persulphate (APS, 99%, Aladdin) (0.06 M, 1.36 g), silver nitrate (AgNO_3_, 99%, Aladdin), palladium chloride (PdCl_2_, 99%, Aladdin), sodium borohydride (NaBH_4_, 98%, Sinopharm Chemical) and methyl orange (MO, 99%, Aladdin) dye were used. *E. coli* (ATCC 25922) and *S. aureus* (ATCC 25923) were used for antibacterial studies. Deionized water was used to carry out all the reactions. NIPAM has been refined before use for cryo-polymerization. NIPAM monomer was decontaminated by the recrystallization route by dissolving 35 g of NIPAM monomer in 25 mL of benzene at 50 °C.

### 2.2. Synthesis of Pure Cryogel

**Scheme 1:**
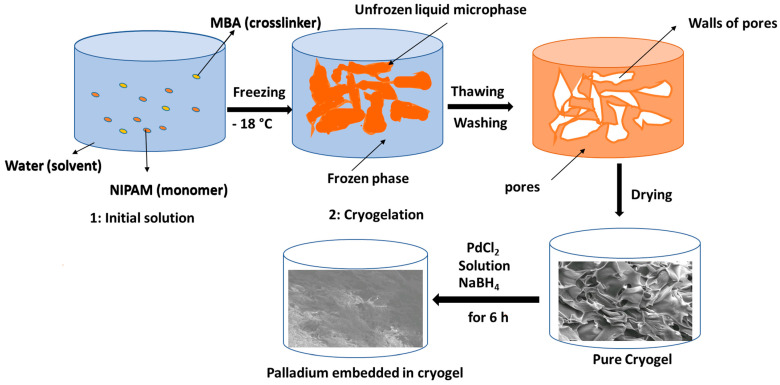
Synthesis scheme of pure NIPAM cryogel and Pd-embedded hybrid cryogel.

The cryogel was synthesized according to the method in the literature with a slight modification [40]. Pure polymeric cryogel was synthesized through the cryo-polymerization method using a fresh aqueous solution of APS. The precursors such as monomer (NIPAM) with cross linker MBA were put in a plastic tube and water was added up to 9 mL along with a TEMED solution of 50 µL. The mixture was stirred for 10–15 min in a sonication bath for complete dissolution of the above-mentioned precursors. In the next step, the above mixture and APS solution were kept in a common laboratory fridge while the temperature was set at −18 °C for 5–7 min. After chilling the mixture and APS solution for 5–7 min, the APS solution (1 mL) was instantly added into a plastic tube containing the mixture of monomer and cross-linker. The respective solution mixture was kept for cryo-polymerization up to 24 h. After the completion of cryo-polymerization, the fabricated cryogel was purified from impurities by decantation for 2 days by changing the water twice a day. The synthesized cryogel was collected, dried in a freeze dryer for 24 h and coded as CR and stored for further use. The fabrication route of pure cryogel is displayed in Scheme 1.

### 2.3. Synthesis of Hybrid Cryogels

Silver and palladium-based hybrid cryogels were synthesized by in-situ chemical reduction method using NaBH_4_ as a reducing agent at room temperature.

#### 2.3.1. Synthesis of Silver Based Cryogel

For the synthesis of silver hybrid cryogel, 10 mL of 3 mM aqueous solution of AgNO_3_ was added into 50 mL of beaker having certain amount of dry cryogel of NIPAM and 0.2 g NaBH_4_ was added as a reducing agent. The beaker was covered with the aluminum foil and the mixture was kept at room temperature for 6 h to reduce Ag^+^ ions to Ag_0_ nanoparticles completely. The hybrid cryogel of silver nanoparticles with brown color was taken out and washed with deionized water for several times to remove impurities from the surface of hybrid cryogel. Cryogel was put in water for two days and water was changed twice a day for purification. In the last step, hybrid cryogel was dried in freeze dryer for 24 h and coded as CR-Ag and stored for further experiments.

#### 2.3.2. Synthesis of Palladium Based Cryogel

Palladium based cryogel was also fabricated with the same method as used for silver based cryogel. For the synthesis of palladium based cryogel, 10 mL of 3 mM aqueous solution of PdCl_2_ was added into 50 mL of beaker containing certain amount of dry cryogel of NIPAM and 0.2 g of NaBH_4_ was added as a reducing agent. The beaker containing mixture was covered with aluminum foil and was kept at room temperature for 6 h to reduce Pd^+^ ions to Pd^0^ nanoparticles completely to form hybrid cryogel of palladium. The palladium based cryogel with black color was taken out from the beaker, washed several times with deionized water to remove impurities. The cryogel was put in water for two days and water was changed twice a day for purification. At last, palladium based cryogel was dried in freeze dryer for 24 h and coded as CR-Pd and kept for further experiments. The fabrication route of silver and palladium hybrid cryogels is displayed in Scheme 1.

### 2.4. Synthesis of Hydrogel and Its Hybrid with Silver Nanoparticles

Hydrogel was prepared by following the same route as used for synthesis of cryogels but polymerization process was carried out at room temperature for 24 h. After 24 hours’ sample was purified with water, dried in oven, and stored for further use. Similarly, hybrid hydrogel was also prepared by same method used earlier for the hybrid cryogels of silver and palladium coded as HG for pure and HG-Ag for hybrid hydrogel. The sample of hydrogel was prepared for comparison of degradation efficiency with cryogel.

### 2.5. Catalytic Study

The prepared hybrid cryogels were used as catalyst for the degradation of an organic dye; methyl orange (MO). Then 0.16 g of methyl orange dye was dissolved in 500 mL of water and stirred to form homogeneous solution. 0.1 g of reducing agent: NaBH_4_ and 0.1 g of catalyst was added to 50 mL of prepared solution of methyl orange. The degradation process was observed by UV-visible spectroscopy. Effect of temperature and amount of catalyst were also observed on degradation rate by varying the temperature from 25 °C to 40 °C. The value of apparent rate constant (k_app_) for the degradation studies has been obtained from the slope of the plot of ln (C_t_/C_0_) versus time by using the following kinetic rate equation.

(1) $$\ln(\frac{C_{t}}{C_{o}})=-k_{\mathrm{app}}\cdot t$$

where, C_t_ is the concentration at time (t), C_o_ is the concentration at time = 0 and k_app_ is apparent rate constant.

### 2.6. Antibacterial Study

By agar well diffusion method, antibacterial activity of pure NIPAM cryogel and its Ag and Pd hybrid cryogel were tested against ATCC (American type culture collection) strains. Test strains of bacteria were Gram-positive *Staphylococcus aureus* (ATCC: 2593) and Gram-negative *Escherichia coli* (ATCC: 25922). These conserved bacterial strains were acquired from microbiology lab at Quaid-i-Azam university, Islamabad and were refreshed on nutrient agar media. This media was prepared, and 24 h fresh bacterial culture was used to make lawn. The procedure comprises of inoculation of bacterial cells on nutrient agar petri dishes and cryogel test samples were laid over these dishes. The cryogel samples in the dishes were incubated at 37 °C for 18–24 h. Subsequently, the growth of bacteria was determined below the cryogel samples. The occurrence of antimicrobial activity was marked by the absence of bacterial growth directly below the test sample.

### 2.7. Instrumentation

The morphology of the cryogels was observed under scanning electron microscopy (SEM, JSM6700F, 10 kV). The test samples were dried pure cryogels sputtered with gold under a vacuum. Fourier transform infrared (FTIR) spectra of cryogels were recorded on a Bruker VECTOR-22 IR spectrometer using KBr pellet samples with a scanning frequency of 4 cm^−1^ and 16 scan times for each wavenumber. The presence of metal nanoparticles in hybrid cryogels was confirmed by X-ray diffraction (XRD, Smart Lab, Rigaku, Beijing Corporation, China). The elemental analysis of silver and palladium hybrid cryogels was observed by using Energy-dispersive X-ray spectroscopy (EDX). UV-visible spectroscopic measurements were performed on a UV-3600 instrument (Shimadzu Scientific Instruments Inc., Columbia, CA, USA).

## 3. Results and Discussion

### 3.1. Scanning Electron Microscopy (SEM) of Cryogels and a Hydrogel

Cryogels are porous materials fabricated under cryogenic conditions via cryo-polymerization. To carryout cryo-polymerization, solubility of precursors in the respective solvent is very important at room temperature as well as at negative temperature. The surface morphology is an important parameter and help to choose the suitable material for its future applications. Cryogels are unique scaffolds having supermacroporous structure that could easily be modulated by changing various parameters like temperature, amount of cross-linker and concentration of precursors etc. [41,42].

**Figure 1:**
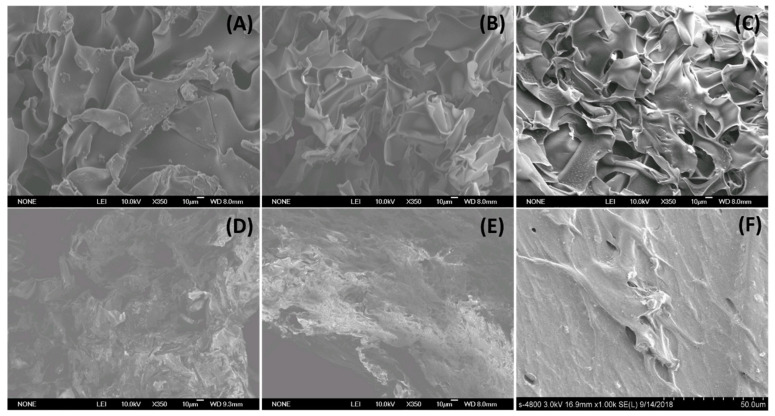
SEM micrographs of NIPAM-based pure cryogels with (**A**) 3% (**B**) 5% and (**C**) 7% crosslinker, (**D**) Ag hybrid cryogels with 3% crosslinker, (**E**) Pd hybrid cryogels with 3% crosslinker and (**F**) NIPAM-based pure hydrogel with 3% crosslinker.

Herein, the effect of cross-linker amount on the surface morphology and porosity of NIPAM based Ag- embedded hybrid cryogels at room temperature has been studied. As displayed in Figure 1, at 3% cross-linker amount (1A), cryogel has few open pores in its network and have got very thin and weak pore wall compared to the cryogels with 5% (1B) and 7% (1C) crosslinker content. For instance, in case of 5% cross-linker, the pores are apparent with thick pore wall and exhibit open pore structure. For cryogel with 7% cross-linker, the pores are very small with very thick pore wall and resembles to a dense polymer material. The surface morphology of hybrid cryogels of silver and palladium has also been analyzed through SEM as shown in Figure 1D,E. For hybrid cryogels; the pore structure cannot be seen very clearly as the nanoparticles may have covered the open pores and make them difficult to be observed. This observation is in accordance with our previous research [40].

Moreover, the comparison of morphology of NIPAM based pure hydrogel with cryogels have also been done. Figure 1F illustrates that the hydrogel has quite dense structure with insignificant porosity in their network structure and this feature mainly differentiate them from cryogels. Despite the pores can be seen as closed, the hybrid cryogels are still porous enough to ensure flow of dye contaminated water. The highly porous structure of the cryogels make them a better candidate for catalytic applications over hydrogel.

### 3.2. Chemical Analysis of Pure and Hybrid Cryogels

**Figure 2:**
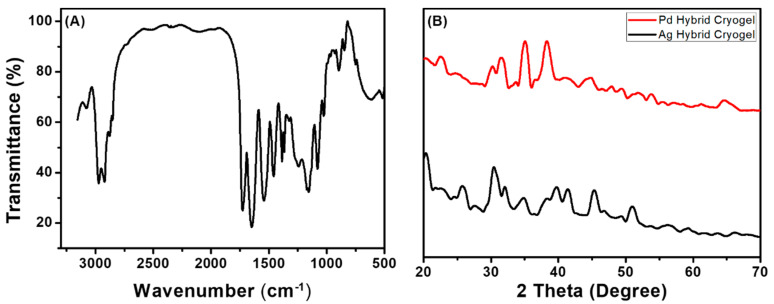
(**A**) FTIR spectrum of NIPAM-based pure cryogels (CR) and (**B**) XRD spectra of Pd-hybrid (CR-Pd) and Ag-hybrid (CR-Ag) NIPAM-based cryogels.

FTIR technique was implemented for the functional group analysis of pure cryogel in the range of 500–4000 cm^−1^ as shown in Figure 2A. Cryopolymerization was confirmed by the disappearance of characteristic band of vinyl group from 3000–3100 cm^−1^ that corresponds to -CH of olefin. The signals around 1652 cm^−1^ and 1548 cm^−1^ are mainly attributed to stretching vibration of amido carbonyl group (N-C=O) and (C=N) groups respectively confirming the successful synthesis of pure NIPAM cryogel [43,44].

For the confirmation of Ag and Pd nanoparticles in the hybrid cryogels, powder XRD analysis was performed, as shown in Figure 2B. The peaks around 31.71°, 44.11° and 64.86° degrees confirm the presence of Pd nanoparticles in the palladium hybrid cryogel (CR-Pd). Similarly, the peaks around 37.85°, 44.11°, 49.65° and 64.50° degrees confirm the presence of silver nanoparticles in the silver hybrid cryogels (CR-Ag). All the respective reflections of the silver hybrid cryogels were indexed as (111), (220) and (211), and for the Pd hybrid cryogels were indexed as (211), (200), (220) and (311), respectively, which are in good agreement with the values in the literature [15]. The particle size is also calculated by using the Scherrer formula and the size was in the range of 40–50 nm.

(2) $$D_{hkl}={\frac{K\lambda }{Bcos\theta }}_{B}$$

where *D_hkl_* is the crystallite size in the direction perpendicular to the lattice planes, hkl are the Miller indices of the planes being analyzed, *K* is a numerical factor frequently referred to as the particle-shape factor, *λ* is the wavelength of the X-rays, *B_hkl_* is the width (full-width at half-maximum) of the X-ray diffraction peak in radians and *θ* is the Bragg angle.

**Figure 3:**
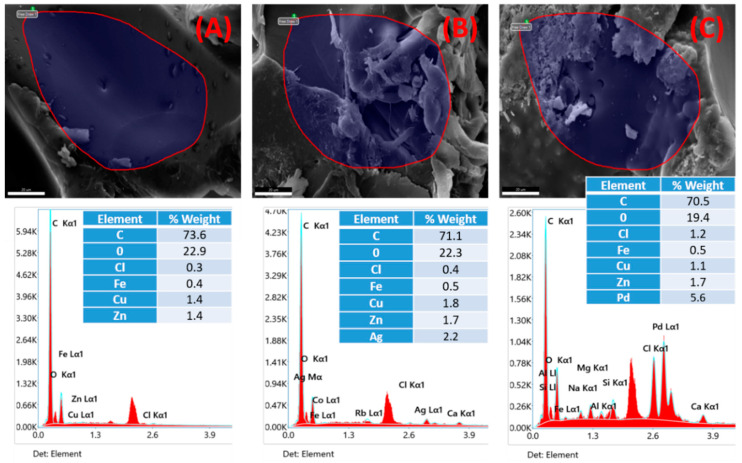
Elemental analysis of (**A**) pure NIPAM-based cryogel (**B**) silver nanoparticles embedded hybrid cryogel and (**C**) palladium nanoparticles embedded hybrid cryogel.

The elemental analysis of pure and fabricated hybrid cryogels were done by using the EDX technique as shown in Figure 3. The EDX analysis has confirmed the presence of silver and palladium nanoparticles in the fabricated hybrid cryogels. The Figure 3A showed the presence of main elements of the monomer and cross-linker present in the backbone of pure cryogel. It could be seen from the table of elements that the palladium content is more as compared to silver. The main reason of greater content of palladium could be attributed to its small size which facilitates its better loading inside the cryogel network. The trend of embedding of Pd and Ag nanoparticles in cryogel matrix is in good agreement with our previous work on NIPAM- based microgels [15].

## 4. Application of Hybrid Cryogels

### 4.1. Degradation of Methyl Orange via Ag Embedded Hybrid Cryogel

Here, the catalytic activity of synthesized *NIPAM*-based hybrid cryogels has been evaluated for the degradation of methyl orange (MO) dye using NaBH_4_ as a reducing agent. The apparent rate constant rate was calculated by plotting ln (C_t_/C_0_) against time. In this reaction, the amount of NaBH_4_ was in excess and the kinetic rate of reaction was considered as pseudo first order. MO give the maximum absorption peak around 464 nm and have reddish orange color in acidic media. When silver-based cryogels were employed as catalysts and NaBH_4_ as a reducing agent, the absorption started to decrease with time and after a certain time it completely disappeared, and the colored solution of dye become colorless, which is the main indication of the degradation of MO dye.

#### 4.1.1. Effect of Temperature on Catalytic Activity of Ag Embedded Hybrid Cryogel

Initially, the degradation was studied at room temperature (25 °C) using the cryogel catalyst dose of 0.05 g and 0.1 g of NaBH_4_. The degradation was completed in 26 min at room temperature for which the apparent rate constant (Kapp) value was 0.1012 min^−1^. The effect of temperature on degradation of dye was further analyzed at different temperatures ranging from 30 °C to 40 °C. As PNIPAM is a thermo-sensitive polymer and has been used for the stabilization of different nanoparticles [45]; therefore, NIPAM based hybrid cryogels are expected to respond to external temperature. The variation in temperature of the reaction medium can affect the degradation rate that could be attributed to the shrinking in NIPAM based cryogel network.

**Figure 4:**
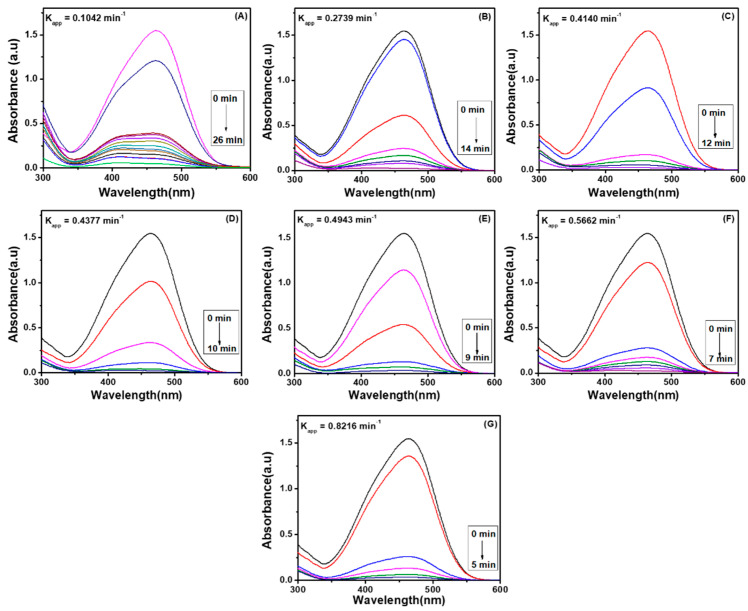
UV-Visible spectra for degradation of Methyl orange (dye) and its apparent rate constant values by using Ag-embedded NIPAM-based cryogels (CR-Ag) at different temperatures: (**A**) 25 °C (**B**) 30 °C (**C**) 31 °C (**D**) 32 °C (**E**) 33 °C (**F**) 35 °C (**G**) 40 °C.

The degradation studies at different temperatures and their Kapp values were calculated for each temperature as shown in Figure 4. With the enhancement of temperature from 25 °C to 30 °C; the Kapp value increases from 0.1012 min^−1^ to 0.2739 min^−1^ which is in accordance with the common observation of molecular kinetic theory which states that with increase in temperature, rate of reaction will increase. However, in this case further enhancement in temperature from 30 °C to 31 °C and 32 °C the K_app_ values enhanced more than usual i.e., 0.4140 min^−1^ and 0.4377 min^−1^ respectively and the reason might be the phase transition behavior of NIPAM. At this stage, the cryogel shrinks and nanoparticles are more exposed towards the surface and reactant molecules react more efficiently [46] resulting in prominent degradation of dye as illustrated in in Figure 4B–D. With further increase in temperature to 33 °C, 35 °C and 40 °C; the Kapp values increases to 0.4943 min^−1^, 0.5662 min^−1^ and 0.8216 min^−1^ respectively, which is the common trend of most of the chemical reactions implying that with the increase in temperature, the rate of reaction increases and vice versa as shown in Figure 4E–G. In addition, it might be due to shrinking of cryogel at higher temperature where surface to volume ratio might have increased, therefore causing the increase in rate constant value. Our designed hybrid cryogel catalyst degradation performance is quite better than other reported hybrid microgels [47]. Mostly gold nanoparticles have been widely embedded in microgel matrix for degradation of different dyes [48,49,50,51]. The cryogel catalyst having macroporous structure embedded with less expensive metal nanoparticles in them make them a better scaffold for catalytic applications.

The effect of temperature variation on the reduction rate has been obtained by Arrhenius equation according to Equation (3) [52]. To investigate the effect of temperature on the methyl orange dye degradation rate, experiments were performed at different temperatures varying from 25 °C to 40 °C. With increasing temperature from 25 °C to 40 °C, the degradation rate constant increased from 0.1042 to 0.8216 min^−1^ respectively.

(3) $$\ln K=\mathrm{Aexp}-(\frac{E_{a}}{RT})$$

**Figure 5:**
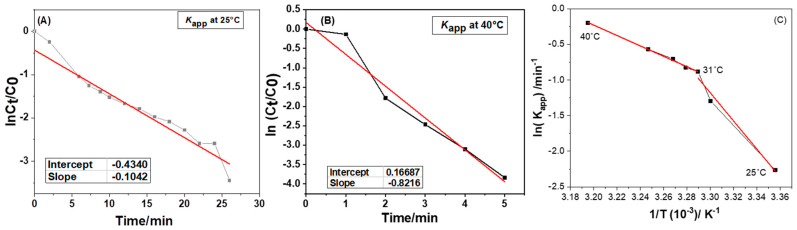
Pseudo-first order kinetic plots for reduction of methyl orange by using Ag-embedded NIPAM based cryogels (CR-Ag) at different temperatures: (**A**) 25 °C (**B**) 40 °C (**C**) Arrhenius plot for temperature dependence of degradation rate.

Arrhenius plot has been used for the calculation of Activation Energy (Ea) at various temperatures by plotting ln k (rate constant) versus 1/T (kelvin) as shown in Figure 5C. Generally,the graph between ln k and 1/T is a straight line with an intercept of ln A and the slope of the graph is equal to −E_a_/R, where R is a constant equal to 8.314 J/mol-K. According with Arrhenius plot (Figure 5C), the activation energy values of 164 kJ/mol has been obtained for degradation of methyl orange dye by using Ag embedded in crygel catalyst before volume phase transition temperature. After 31 °C, the slope changes and the activation energy value comes out to be 60 kJ/mol. The low activation energy after phase transition indicates that the dye degradation process may have become thermodynamically feasible. The reason of the increase in rate constant of reduction process may correspond to more probable conformation of cryogel in solution with more exposed catalytic sites. However, the complete correspondence between phase transition behaviour of hybrid cryogel catalyst and rate of reaction requires a systematic study in future.

#### 4.1.2. Effect of Dosage of Catalyst on Degradation Performance of Ag Embedded Hybrid Cryogels

**Figure 6:**
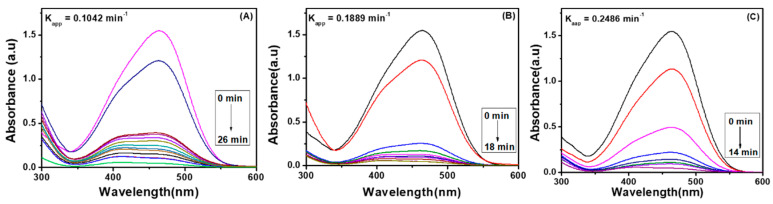
UV-Visible spectra for degradation of Methyl orange (dye) and its apparent rate constant values by using Ag-embedded NIPAM-based cryogels (CR-Ag) at different cryogel catalyst amounts: (**A**) 0.05 g (**B**) 0.10 g (**C**) 0.15 g.

After evaluating the effect of temperature, the effect of amount of silver-based cryogel catalyst on the degradation performance for methyl orange was also studied. The amount (0.1 g) of reducing agent as well as the amount (0.32 mg/mL) of the dye (MO) were kept constant throughout the experiment. At lower catalyst (i.e., Ag embedded in cryogel) concentration (0.05 g); the degradation was completed in 26 min with K_app_ value of 0.1012 min^−1^ as shown in Figure 6A. By increasing the amount of catalyst to 0.10 g, the catalytic reduction took 18 min with K_app_ value of 0.1889 min^−1^ as illustrated in Figure 6B. With further enhancement of catalyst amount (0.15 g), the reduction occurred in 14 min with K_app_ value of 0.2489 min^−1^ as depicted in Figure 6C. The results revealed that the catalyst amount has a pronounced effect on the degradation performance as shown in Figure 6. The enhancement of the degradation is attributed to the availability of more active sites in case of higher amount of Ag loaded cryogel catalyst as compared to lesser amount. The value of Kapp was calculated by plotting ln (C_t_/C_0_) vs. time.

### 4.2. Degradation of Methyl Orange via Pd Embedded Hybrid Cryogel

**Figure 7:**
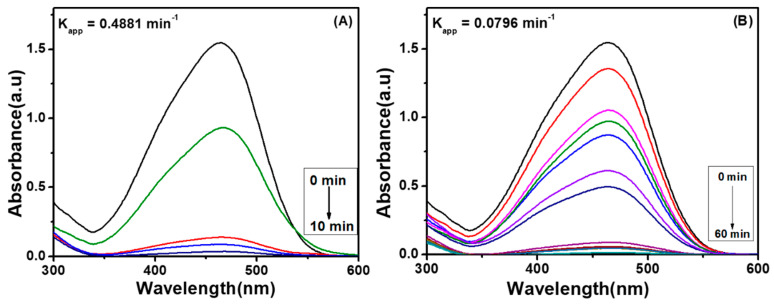
UV-Visible spectra for degradation of Methyl orange (dye) and its apparent rate constant values at 25 °C by using; (**A**) Pd-embedded NIPAM based cryogel (CR-Pd) (**B**) Ag-embedded NIPAM based hydrogel (HG-Ag).

After the detailed study of the silver-based catalyst, the catalytic performance of palladium-embedded in cryogel catalyst was also studied at room temperature (25 °C). The palladium-based catalyst was also employed with the same methodology as already implemented for silver- based catalyst. The amount of reducing agent and amount of methyl orange dye was kept constant i.e., 0.05 g and 0.32 mg/mL respectively. In case of palladium-based catalyst the reduction was completed in 10 min at room temperature with K_app_ value of 0.4881 min^−1^ (Figure 7A), which is much efficient compared to silver- based catalyst as illustrated in Figure 4A. The better degradation performance of CR-Pd catalyst may be attributed to its smaller size compared to silver-embedded in cryogel catalyst. In our previous report, it has already been mentioned that the small sized nanoparticles have more surface area as compared to large sized nanoparticles and in turn showed better catalytic performance [15].

### 4.3. Comparative Study of Catalytic Performance of Fabricated Hybrid Cryogels with Ag-Hybrid Hydrogel for Degradation of Methyl Orange

For the comparative study, hybrid hydrogel of silver was fabricated via reduction method and its reduction performance against methyl orange was evaluated as displayed in Figure 7B. From the results of apparent rate constant value, it is quite clear that hybrid hydrogel of silver is very slow in reduction of MO (Figure 7B) in comparison with silver embedded in cryogel (Figure 4A). The reduction was completed in almost 60 min with K_app_ value of 0.0796 min^−1^ for hydrogel, which is quite low compared to Ag-embedded in cryogel where degradation took 26 min with K_app_ value of 0.1042 min^−1^ as already depicted in Figure 4A. The main reason of low reduction performance could be attributed to the closed cavities and dense structure of hydrogel which is also in good agreement with SEM image (Figure 1F) where no open pores could be clearly observed.

### 4.4. Antibacterial Activities of Pure NIPAM Cryogel and Silver (Ag) and Palladium (Pd) Embedded Hybrid Cryogels

**Figure 8:**
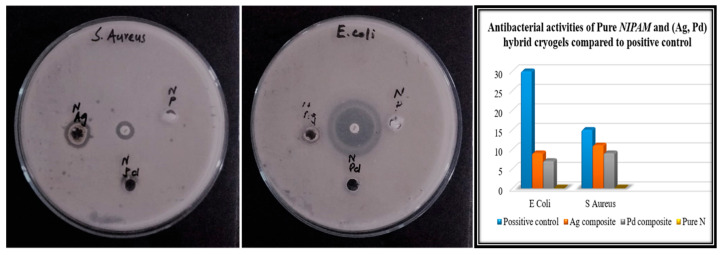
Antibacterial activities of pure NIPAM cryogel (CR), Ag-embedded NIPAM-based cryogels (CR-Ag) and Pd-embedded NIPAM-based cryogels CR-Pd against *Staphylococcus aureus* and *Escherichia coli*.

The antibacterial activity of pure *NIPAM* cryogel and its Ag and Pd hybrid cryogels were tested against ATCC (American type culture collection) strains. Two test strains of bacteria used were: Gram-positive *Staphylococcus aureus* (ATCC: 2593) and Gram-negative *Escherichia coli* (ATCC: 25922). All the cryogels samples i.e., pure, Ag, and Pd hybrid were tested against ATCC bacterial strains by agar well diffusion method. After 24 h of incubation, it was observed that hybrid cryogels gave maximum zone of inhibition against *Staphylococcus aureus* and *Escherichia coli* as shown in Figure 8. On the contrary, *NIPAM*-based pure cryogel has no zone of inhibition against both bacterial strains. However, *NIPAM* cryogels with their silver hybrid has greater zone of inhibition against both positive and negative bacterial strains as compared to Pd hybrid cryogels. Ag-based composite materials have already been used as antimicrobial agent [53] Antibacterial activity of pure and hybrids cryogels are shown in Figure 8. The Table 1 also illustrates the diameter of zone of inhibition for each bacterium in detail. Both hybrid cryogels are quite effective against gram -positive and gram-negative bacteria.

## 5. Conclusions and Prospects

Facile synthesis of *NIPAM* based pure cryogel and its hybrid cryogels were carried out by cryo-gelation and chemical reduction method respectively. The cross-linker content effect on porosity of Ag- embedded in cryogels was evaluated. These fabricated hybrid cryogels were employed for efficient degradation of methyl orange dye. The degradation studies showed that the palladium catalyst was more efficient owing to its small size as compared to silver catalyst for degradation of organic dye at room temperature. Effect of temperature on the catalytic activity of silver hybrid cryogels was also studied. High temperature was more efficient for the degradation of dye as compared to lower temperature. The catalyst dose rate was also examined, and results confirmed that higher concentration have shown better results as compared to lower concentration. Finally, both the hybrid cryogels have also shown good antibacterial activities as compared to pure cryogel. The presence of Ag and Pd nanoparticles in cryogel matrix render them significant antibacterial properties and performance enhancement. Therefore, our fabricated dual purpose cryogel catalyst is a preferable choice for systematic studies in multiple applications. To conclude, this study could provide the sufficient basis for devising better and cost-effective catalysts in future for water purification and removing the toxicity of dyes from contaminated water. The fabrication is very efficient, economical and can be implemented on industrial scale in near future.

## Figures and Tables

**Table 1:** Zone of inhibition of both *E. coli* and *S. aureus*.

Samples	ATCC Bacterial Strains	Zone of Inhibition
Pure NIPAM	*Staphylococcus aureus*	No zone of inhibition
NIPAM with Ag hybrid	*Staphylococcus aureus*	11 mm
NIPAM with Pd hybrid	*Staphylococcus aureus*	9 mm
Positive control	*Staphylococcus aureus*	15 mm
Pure NIPAM	*Escherichia coli*	No zone of inhibition
NIPAM with Ag hybrid	*Escherichia coli*	9 mm
NIPAM with Pd hybrid	*Escherichia coli*	7 mm
Positive control	*Escherichia coli*	30 mm

## Data Availability

Not applicable.
